# Acylation of Heteroaromatic Amines: Facile and Efficient Synthesis of a New Class of 1,2,3-Triazolo[4,5-*b*]pyridine and Pyrazolo[4,3-*b*]pyridine Derivatives

**DOI:** 10.3390/molecules16053723

**Published:** 2011-05-04

**Authors:** Hamada Mohamed Ibrahim, Haider Behbehani, Saad Makhseed, Mohamed H. Elnagdi

**Affiliations:** 1 Chemistry Department, Faculty of Science, Kuwait University, P.O. Box 5969, Safat 13060, Kuwait; 2 Chemistry Department, Faculty of Science, Fayoum University, Fayoum, A. R., Egypt

**Keywords:** cyanoacetic acid, cyanoacetamides, triazolo[4,5-*b*]pyridine, pyrazolo[4,3-*b*]-pyridine, * p*-nitrophenylacetic acid, zeolite

## Abstract

1,2,3-Triazolo[4,5-*b*]pyridines and pyrazolo[4,3-*b*]pyridines can be readily prepared *via* cyanoacetylation reactions of 5-amino-1,2,3-triazoles **1a,b** and 4-amino- pyrazole **2** followed by subsequent cyclization of the formed cyanoacetamides. Reactions of amines **1a**,**b** with a mixture of *p*-nitrophenylacetic acid and acetic anhydride under microwave irradiation conditions afforded the corresponding amides **15a,b** that underwent cyclization to form 1,2,3-triazolo[4,5-*b*]pyridines **16a,b** upon heating in DMF solutions containing sodium acetate. Reactions of **1a,b** with active methylene compounds, including **17a-c**, in the presence of zeolites as catalyst also afforded 1,2,3-triazolo[4,5-*b*]pyridine derivatives **20a-f** *via* the intermediacy of triazole derivatives **19** and not **18**.

## 1. Introduction

Pyrazolo[4,3-*b*]pyridine and triazolo[4,5-*b*]pyridine derivatives are of interest for their various applications as vasodilators, hypotensive, hypoglycemic, anti-inflammatory, analgesic, antiasthmatic, antipyretic agents and as substrates of NAD glycohydrolase [1,2,3]. Owing to these interesting biological activities and medicinal properties these azolopyridine derivatives have been the targets of investigations by several research groups [4,5,6,7,8]. The present study describes the results of an investigation aimed at the preparation of a new class of 1,2,3-triazolo[4,5-*b*]pyridine and pyrazolo[4,3-*b*]pyridine derivatives. Cyanoacetylation of electron rich aromatic compounds and heteroarmatic amines, initially described by Slatt *et al.*, [9] has found extensive utility in efficient routes for the preparation of 3-oxoalkanonitriles [10,11,12,13,14,15] and cyanoacetamides [16,17,18]. Cyanoacetylation of *o*-acyl heteroarmatic amines is expected to give cyanoacetamides in which an active methylene moiety is located in close proximity to a ketone carbonyl function. This enables ready cyclization of the products to form fused pyridines. However, to our knowledge this synthetic approach has not been explored to date. Below, we describe the results of an investigation of the preparation of cyanoacetamides and arylacetamides of 5-amino-1,2,3-triazoles and 4-aminopyrazoles and their utility in the preparation of condensed pyridines.

## 2. Results and Discussion

**Scheme 1:**
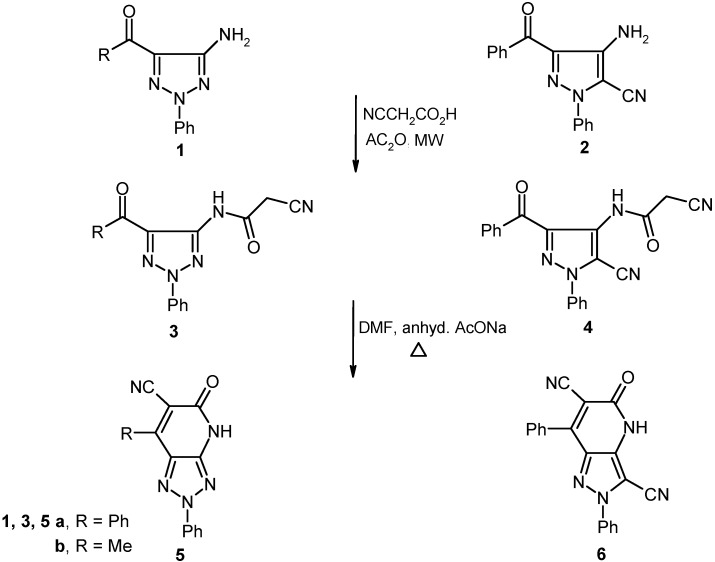
Reaction of 5-amino-1,2,3-triazoles and 4-aminopyrazole with cyanoacetic acid.

**Figure 1:**
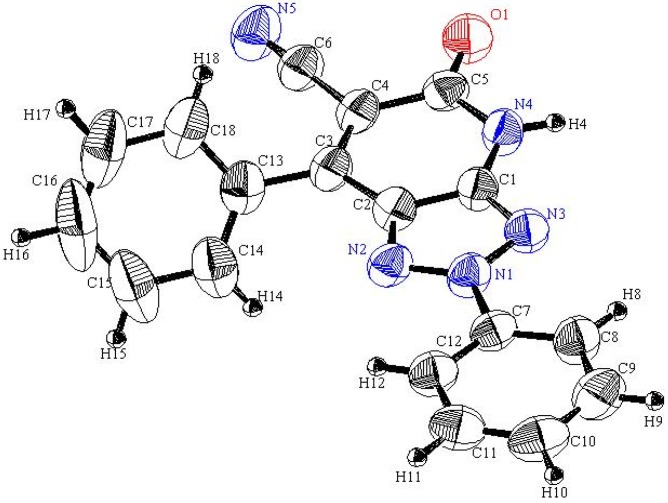
ORTEP plot of the x-ray crystallographic data determined for **5a**. Crystallo- graphic data have been deposited with the Cambridge Crystallographic Data Centre as supplementary publication number CCDC 804721 [22].

Readily obtainable (5-amino-2-phenyl-*2H*-1,2,3-triazol-4-yl)phenylmethanone (**1a**) [19], 1-(5-amino-2-phenyl-*2H*-1,2,3-triazol-4-yl)ethanone (**1b**) [20] and 4-amino-3-benzoyl-1-phenyl-1*H*-pyrazole-5-carbonitrile (**2**) [21] were found to react with a preheated mixture of acetic anhydride and cyanoacetic acid under microwave irradiation conditions to yield the corresponding cyanoacetamides **3** and **4** in excellent yields. These substances undergo cyclization to generate the respective fused pyridones **5** and **6** upon stirring at reflux for 30 min in DMF containing anhydrous sodium acetate (cf. Scheme 1). The structure of **5a** was assigned by using X-ray crystallographic analysis (cf. Table 1 and Figure 1).

**Table 1:** Selected bond lengths and bond angles for **5a**.

Bond	Bond length	Bond	Bond angle
N1-N2	1.327	N1-N2-C2	102.5
N1-N3	1.356	N1-N3-C1	100.4
N3-C1	1.333	N2-N1-N3	117.5
N2-C2	1.350	N3-C1-C2	111.6
C1-C2	1.384	N2-C2-C1	108.0
C1-N4	1.375	C1-C2-C3	121.4

The acylpyrazole **7** underwent ready cyanoacylation to afford the cyanoacetamide **8** in 93% yield. Heating a solution of **8** in DMF containing anhydrous sodium acetate leads to production of a substance whose structure should be either **9** or its isomer **10**. 

**Scheme 2:**
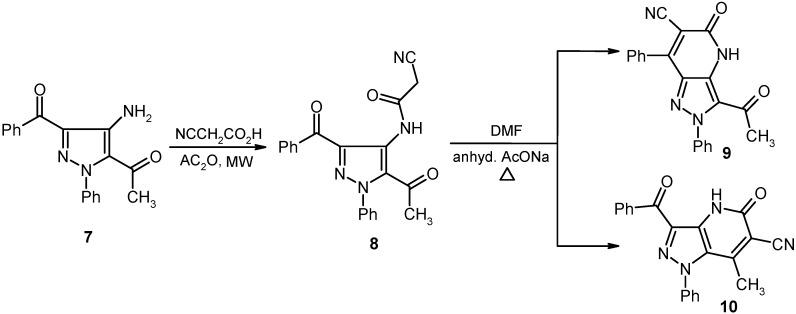
Synthesis of pyrazolo[4,3-*b*]pyridine-6-carbonitrile **10**.

**Figure 2:**
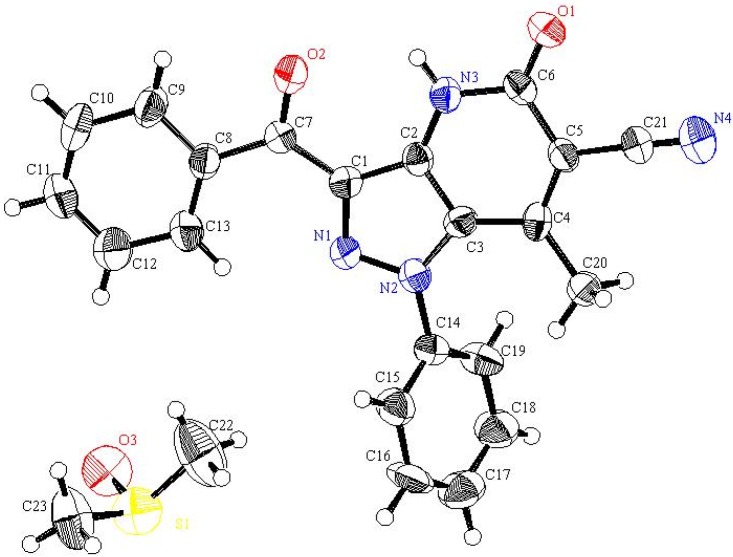
ORTEP plot of the x-ray crystallographic data determined for **10** containing one DMSO molecule. Crystallographic data have been deposited in the Cambridge Crystallographic Data Centre as supplementary publication number CCDC 816562 [23].

The actual structure of the product was assigned as 10 based on its ^13^C-NMR spectroscopic data which showed the absence of an acetyl carbonyl carbon resonance and its replacement by a peak at 186.19 ppm. Moreover, the methyl protons’ resonance at *δ* = 2.12 ppm displays a HMBC cross peak with the carbon peak at *δ* = 105.72 ppm that is assigned as C-6. In addition the X-ray crystallographic analysis of this product demonstrated that it has the structure represented by 10 (cf. Scheme 2 and Figure 2).

**Scheme 3:**
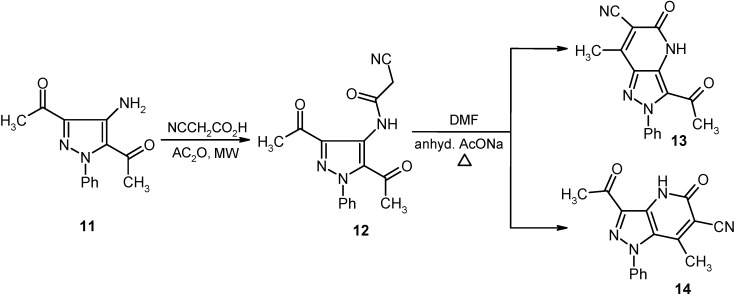
Synthesis of pyrazolo[4,3-*b*]pyridine-6-carbonitrile **14**.

In a similar manner, bis-acetylpyrazole **11** is readily cyanoacylated to afford the corresponding cyanoacetamide **12** in excellent yield. Heating a DMF solution of **12** containing anhydrous sodium acetate afforded a product that may also have the isomeric structures represented by **13** and **14**. As before, the actual structure of the product was shown to be **14** based on its ^13^C-NMR spectrum, which contained a carbonyl resonance at 202.41 ppm. This chemical shift is expected for an acyl carbonyl at the C-3 position of the pyrazole ring and not at C-5 since in the latter case shielding provided by the N-lone pair should make the resonance appear at a higher field (cf. Scheme 3).

**Scheme 4:**
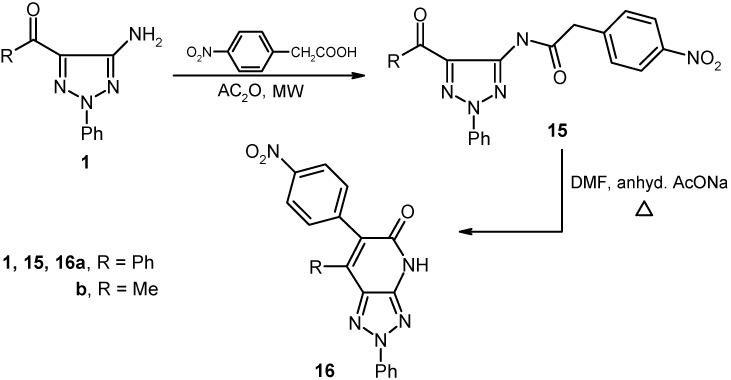
Reaction of 5-amino-1,2,3-triazoles with *p*-nitrophenylacetic acid.

We have previously [10] suggested that in the mechanistic pathway for the process described by Slatt [9], the mixed anhydride is formed initially and then it reacts by nucleophilic addition of an amine or electron rich aromatic system at the more electron deficient cyanoacetyl carbonyl. As a consequence of this proposal, we believed that other mixed anhydrides could be used as arylacetamide precursors provided that the reactions occur at the more electron deficient aroyl carbonyl. In fact, heating *p*-nitrophenylacetic acid with acetic anhydride, followed by addition of either **1a** or **1b** and heating the mixture in a microwave oven for 60 s, afforded the corresponding amides **15a** and **15b** that are readily cyclized to form the respective triazolo[4,5-*b*]pyridines derivatives **16a** and **16b** upon heating in DMF containing anhydrous sodium acetate (cf. Scheme 4). 

**Figure 3:**
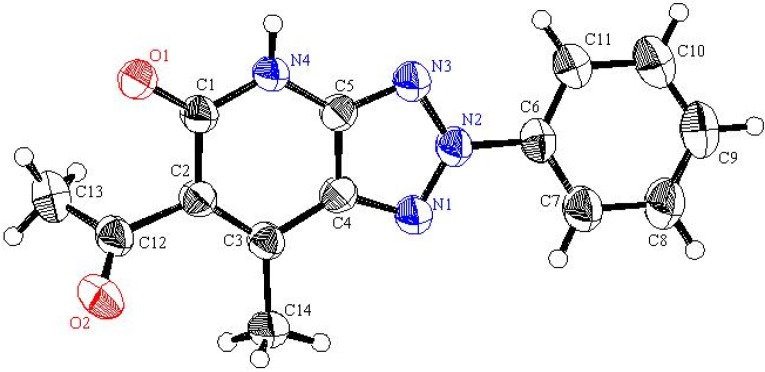
ORTEP plot of the x-ray crystallographic data determined for **20f**. Crystallo- graphic data have been deposited in the Cambridge Crystallographic Data Centre as supplementary publication number CCDC 815380 [24].

**Scheme 5:**
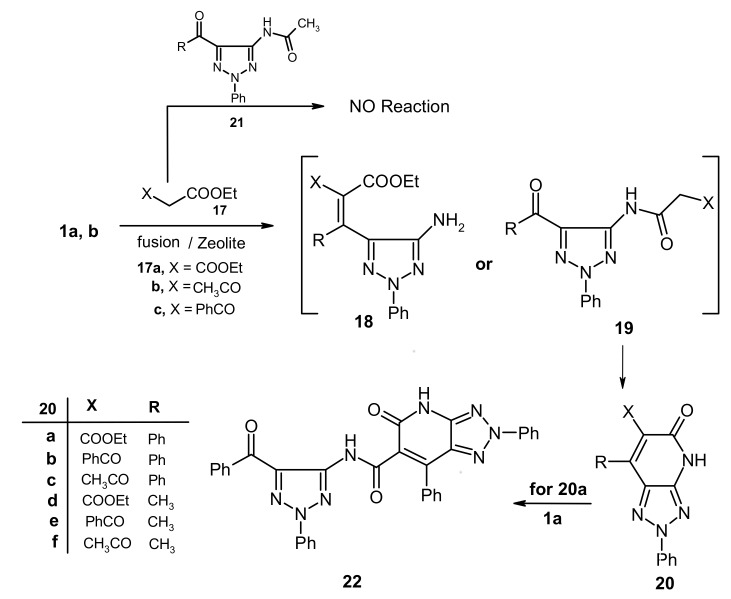
Reaction of 5-amino-1,2,3-triazoles with active methylene compounds.

We have explored a possible extension of this methodology, which relies on conversion of the acetamide derivatives of 1,2,3-triazoles to their corresponding 1,2,3-triazolo[4,5-*b*]pyridines, by probing the reactivity **1a,b** with other active methylene compounds like **17a-c**. The results of this study showed that **1a,b** underwent condensation with **17a-c** in presence of zeolite catalysts, followed by heating to produce the corresponding 1,2,3-triazolo[4,5-*b*]pyridine derivatives **20a-f**. The structure of **20f** was assigned by X-ray crystallographic analysis. Although these products could potentially formed *via* the intermediacy of either triazole **18** or **19**, it is almost certain that **19** is the intermediate as attempts to condense *N*-acetyl-1,2,3-triazole derivatives **21** with active methylene compounds failed. Moreover the reaction of **20a** with another molecule of **1a** afforded **22**, which is generated *via* elimination of ethanol (cf. Figure 3 and Scheme 5).

**Scheme 6:**
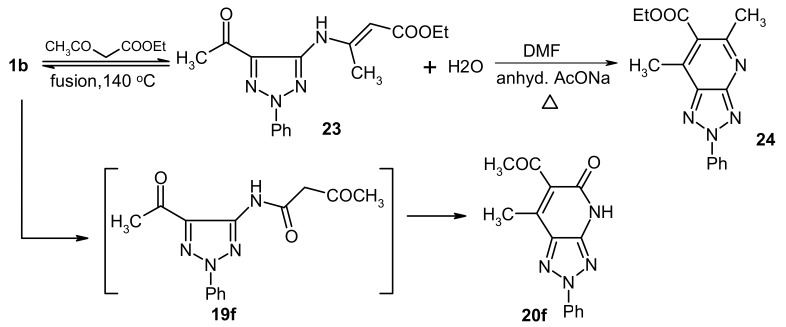
Reaction of **1b** with ethyl acetoacetate to afford **24**.

**Figure 4:**
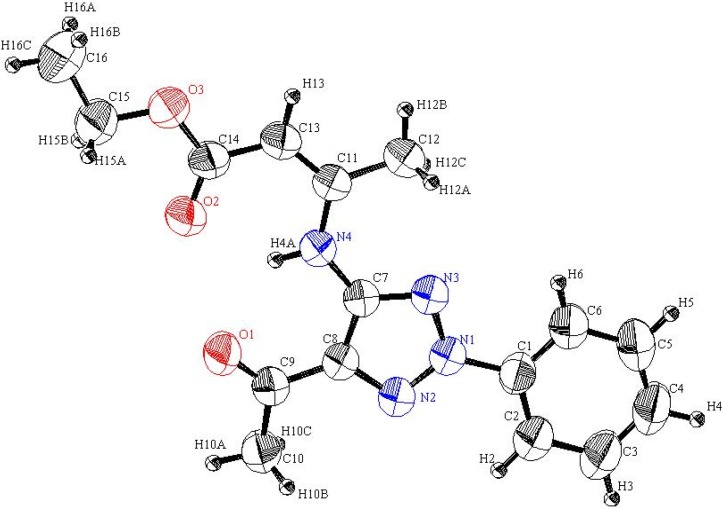
ORTEP plot of the x-ray crystallographic data determined for **23**. Crystallo- graphic data have been deposited in the Cambridge Crystallographic Data Centre as supplementary publication number CCDC 805282 [25].

In contrast, **1b** was found to react with ethyl acetoacetate (**17b**) in absence of zeolite to yield a condensation product that arises by elimination of one molecule of water. X-ray crystallographic analysis of this substance demonstrated that it has the structure represented by **23**, a product that is formed *via* initial addition of the amine to the carbonyl carbon of **17b** (Figure 4, Table 2).The fact that **1b** reacts with ethyl acetoacetate (**17b**) to yield either the intermediate **23** or **19f** demonstrates the effect of the zeolite, a microporous catalyst that favors formation of slim molecules like **19** rather than bulky ones like **23**, so the latter is formed in absence of such **a** catalyst, Also, **23** separated from the reaction mixture underwent cyclization in refluxing DMF containing anhydrous sodium acetate to form **24** *via* loss of another molecule of water. (cf. Scheme 6).

**Table 2:** Selected bond lengths and bond angles for **23**.

Bond	Bond length	Bond	Bond angle
N1-N2	1.317	N1-N2-C8	103.8
N1-N3	1.359	N1-N3-C7	102.7
N3-C7	1.330	N2-N1-N3	116.1
N2-C8	1.336	N3-C7-C8	109.0
C8-C9	1.462	N2-C8-C7	108.5
N4-C7	1.379	O1-C9-C8	119.5

Inspection of the crystallographically determined bond angles and lengths of the 1,2,3-triazole rings in both **5a**, **20f** and **23** indicate that the N3-N1-N2 bond angles deviate significantly from typical sp^3^ nitrogen values and are close to those that are associated with sp^2^ nitrogens. However, the N2-C8-C7 or N3-C7-C8 bond angles are close to those expected for sp^3^ carbons. Similar observations, made earlier in studies of very similar systems by Elnagdi *et al.*, [19] have been taken as evidence for the significant contribution of charge separated resonance forms delocalizing N-1 lone pairs to the ring carbons. Importantly, both **5a**, **20f** and **23** are planar substances, a fact that adds further support to the conclusion that resonance delocalization of N-1 lone pair occurs in these systems.

## 3. Experimental

### 3.1. General

Melting points were recorded on a Griffin melting point apparatus and are reported uncorrected. IR spectra were recorded using KBr disks using a Perkin-Elmer System 2000 FT-IR spectrophoto- meter. ^1^H-NMR (400 MHz) and ^13^C-NMR (100 MHz) spectra were recorded at 25 °C in CDCl_3_ or DMSO-*d_6_* as solvent with TMS as internal standard on a Bruker DPX 400 super-conducting NMR spectrometer. Chemical shifts are reported in ppm. Mass spectra were measured using a high resolution GC-MS (DFS) thermo spectrometers with EI (70 EV). Microanalyses were performed on a LECO CHNS-932 Elemental Analyzer. Reactions were conducted under microwave irradiation in heavy-walled Pyrex tubes (capacity 10 mL) fitted with PCS caps. Microwave heating was carried out with a single mode cavity Explorer Microwave synthesizer (CEM Corporation, 3100 Smith Farm Road, Matthews, NC, USA). The zeolite (≤ 45 µm) was purchased from Fluka Company with product No. 96096. The crystal structures were determined by a Rigaku R-AXIS RAPID diffractometer using filtered Mo-Kα radiation at Kuwait University. Compounds **1a,b, 2** and **21** were prepared using literature procedures [19,20,21].

### 3.2. General Procedure for the Preparation of Cyanoacetamides ***3***, ***4***, ***8*** and ***12***

A solution of cyanoacetic acid (0.45 g, 5 mmol) in Ac_2_O (5 mL) was heated in the microwave oven at 85 °C for 10 s then compounds **1**, **2**, **7** or **11** (5 mmol) were added and the reaction mixture was heated for further 30 s at 100 °C. The reaction mixture was allowed to cool to room temperature and the formed crystalline solid was separated by filtration and washed with cold ethanol and then hot ethanol to afford **3, 4, 8** and **12**, respectively, as pure substances.

*N-(5-Benzoyl-2-phenyl-2H-1,2,3-triazol-4-yl)-2-cyanoacetamide* (**3a**). Creamy white crystals, yield: 98%, m.p. 210 °C; IR (KBr): ν/cm^−1^ 3291 (NH), 2257 (CN), 1692, 1634 (2CO); ^1^H-NMR (DMSO-*d*_6_): δ = 4.04 (s, 2H, CH_2_), 7.50 (t, *J* = 7.6 Hz, 1H, Ar-H), 7.56–7.64 (m, 4H, Ar-H), 7.73 (t, *J* = 7.6 Hz, 1H, Ar-H), 8.01 (d, *J* = 7.6 Hz, 2H, Ar-H), 8.10 (d, *J* =7.6 Hz, 2H, Ar-H) and 11.23 ppm (s, 1H, NH); ^13^C-NMR (DMSO-*d*_6_): δ 26.03 (CH_2_), 115.35 (CN), 118.76, 128.64, 128.68, 129.91, 129.97, 133.77, 136.04, 137.22, 138.48, 144.19, 161.40 and 185.82 ppm (Ar-C and CO); MS (EI): m/z (%) 331 (M^+^, 74.35), 332 (M^+^+1, 16.90). Anal. calcd. for C_18_H_13_N_5_O_2_ (331.34): C, 65.25; H, 3.95; N, 21.14. Found: C, 65.28; H, 4.02; N, 21.20.

*N-(5-Acetyl-2-phenyl-2H-1,2,3-triazol-4-yl)-2-cyanoacetamide* (**3b**). Buff crystals, yield: 95%, m.p. 205 °C; IR (KBr): ν/cm^−1^ 3302 (NH), 2262 (CN), 1685, 1638 (2CO); ^1^H-NMR (DMSO-*d*_6_): δ 2.62 (s, 3H, CH_3_), 4.11 (s, 2H, CH_2_), 7.51 (t, *J* = 8.0 Hz, 1H, Ar-H), 7.62 (t, *J* = 8.0 Hz, 2H, Ar-H), 8.03 (d, *J* = 8.0 Hz, 2H, Ar-H) and 10.67 ppm (s, 1H, NH); ^13^C-NMR (DMSO-*d*_6_): δ 26.28 (CH_2_), 27.71 (CH_3_), 115.51 (CN), 118.79, 128.85, 129.90, 137.79, 138.46, 143.32, 161.66 and 191.69 ppm (Ar-C and CO); MS (EI): m/z (%) 269 (M^+^, 100), 270 (M^+^+1, 25.6). Anal. calcd. for C_13_H_11_N_5_O_2_ (269.26): C, 57.99; H, 4.12; N, 26.01. Found: C, 58.04; H, 4.06; N, 25.93.

*N-(3-Benzoyl-5-cyano-1-phenyl-1H-pyrazol-4-yl)-2-cyanoacetamide* (**4**). Creamy white crystals, yield: 93%, m.p. 234 °C; IR (KBr): ν/cm^−1^ 3259 (NH), 2227, 2263 (2CN), 1712, 1631 (2CO); ^1^H-NMR (DMSO-*d_6_*, 25 °C): *δ* = 4.13 (s, 2H, CH_2_), 7.57–7.72 (m, 6H, Ar-H), 7.83 (d, *J* = 7.6 Hz, 2H, Ar-H), 8.14 (d, *J* = 7.6 Hz, 2H, Ar-H) and 10.83 ppm (s, 1H, NH); ^13^C-NMR (DMSO-*d_6_*): *δ* 25.84 (CH_2_), 109.64, 110.24, 115.41, 124.20, 128.60, 128.75, 129.76, 130.08, 130.20, 133.74, 135.94, 137.70, 141.38, 161.56 and 186.71 ppm (2CN, Ar-C and CO); MS (EI): m/z (%) 355 (M^+^, 73.20), 356 (M^+^+1, 20.35). Anal. calcd. for C_20_H_13_N_5_O_2_ (355.36): C, 67.60; H, 3.69; N, 19.71. Found: C, 67.57; H, 3.75; N, 19.74.

*N-(5-Acetyl-3-benzoyl-1-phenyl-1H-pyrazol-4-yl)-2-cyanoacetamide* (**8**). Orange crystals, yield: 93%, m.p. above 300 °C; IR (KBr): ν/cm^−1^ 3297 (NH), 22114 (CN), 1687 (br), 1636 (3CO); ^1^H-NMR (DMSO-*d_6_*): *δ* = 2.40 (s, 3H, CH_3_), 4.02 (s, 2H, CH_2_), 7.53–7.60 (m, 7H, Ar-H), 7.69 (t, *J* = 7.2 Hz, 1H, Ar-H), 8.10 (d, *J* = 7.6 Hz, 2H, Ar-H) and 10.45 ppm (s, 1H, NH); ^13^C-NMR (DMSO-*d_6_*): *δ* 25.72 (CH_2_), 29.87 (CH_3_), 115.54 (CN), 122.13, 125.61, 128.48, 129.13, 129.17, 130.14, 133.42, 135.78, 136.26, 139.65, 142.76, 162.14, 187.20 and 189.62 ppm (Ar-C and CO); MS (EI): m/z (%) 372 (M^+^, 30.20), 373 (M^+^+1, 8.57). Anal. calcd. for C_21_H_16_N_4_O_3_ (372.39): C, 67.73; H, 4.33; N, 15.05. Found: C, 67.69; H, 4.31; N, 15.10.

*2-Cyano-N-(3,5-diacetyl-1-phenyl-1H-pyrazol-4-yl)acetamide* (**12**). Creamy white crystals, yield: 89%, m.p. 230 °C; IR (KBr): ν/cm^−1^ 3284 (NH), 2260 (CN), 1685 (br), 1637 (3CO); ^1^H-NMR (DMSO-*d_6_*): *δ* = 2.33 (s, 3H, CH_3_), 2.57 (s, 3H, CH_3_), 4.03 (s, 2H, CH_2_), 7.49–7.57 (m, 5H, Ar-H) and 10.34 ppm (s, 1H, NH); ^13^C-NMR (DMSO-*d_6_*): *δ* 26.16 (CH_2_), 27.72 (CH_3_), 30.22 (CH_3_), 116.01 (CN), 125.95, 127.94, 129.63, 129.68, 137.18, 140.05, 143.17, 162.64, 190.18 and 193.80 ppm (Ar-C and CO); MS (EI): m/z (%) 310 (M^+^, 55.1), 311 (M^+^+1, 10.75). Anal. calcd. for C_16_H_14_N_4_O_3_ (310.31): C, 61.93; H, 4.55; N, 18.05. Found: C, 61.88; H, 4.57; N, 17.98.

### 3.3. General Procedure for the Cyclization of Cyanoacetamides to Azolo Pyridines ***5***, ***6***, ***10*** and ***14***

Independent solutions of cyanoacetamides **3, 4, 8** and **12** (5 mmol), in DMF (10 mL) containing anhydrous sodium acetate (1 g) were stirred at reflux for 1 h. Then, the reaction mixture was cooled to room temperature and poured into ice cold water. The formed crude products were collected by filtration, washed with water and recrystallized from the appropriate solvent to afford the corresponding azolo pyridine derivatives **5, 6, 10** and **14**, respectively.

*5-Oxo-2,7-diphenyl-4,5-dihydro-2H-[1,2,3]triazolo[4,5-b]pyridine-6-carbonitrile* (**5a**). Recrystallized from a EtOH/dioxane (1:1) mixture as yellow crystals, yield: 87%, m.p. above 300 °C; IR (KBr): ν/cm^−1^ 3437 (NH), 2230 (CN), 1651 (CO); ^1^H-NMR (DMSO-*d_6_*): *δ* 7.47 (t, *J* = 7.6 Hz, 1H, Ar-H), 7.56 (t, *J* = 8.0 Hz, 2H, Ar-H), 7.65–7.67 (m, 3H, Ar-H), 7.89 (d, *J* = 7.6 Hz, 2H, Ar-H), 7.97 (d, *J* = 8.0 Hz, 2H, Ar-H) and 13.30 ppm (s, 1H, NH); ^13^C-NMR (DMSO-*d_6_*): *δ* 105.22, 115.73 (CN), 119.10, 128.81, 129.21, 129.53, 129.95, 130.53, 131.14, 131.51, 138.62, 148.07, 151.23 and 160.14 ppm (Ar-C and CO); MS (EI): m/z (%) 313 (M^+^, 100), 314 (M^+^+1, 21.40). Anal. calcd. for C_18_H_11_N_5_O (313.32): C, 69.00; H, 3.54; N, 22.35. Found: C, C, 69.03; H, 3.48; N, 22.39.

#### 3.3.1. Crystallographic Analysis for ***5a***

The crystals were mounted on a glass fiber. All measurements were performed on a Rigaku R-AXIS RAPID diffractometer using filtered Mo-Kα radiation. The data were collected at a temperature of 20 ± 1 °C to a maximum 2θ value of 55.0° using the ω scanning technique. The structure was solved by charge flipping method and expanded using Fourier techniques. The non-hydrogen atoms were refined anisotropically. Hydrogen atoms were refined using the riding model.

#### 3.3.2. Crystal Data

C_18_H_11_N_5_O, M = 313.32, triclinic, a = 6.861(4)Å, b = 11.229(6)Å, c = 12.987(7)Å, V = 869.3(8)Å^3^, α = 110.560(9)°, β = 103.584(9)°, γ = 100.858(9)°, space group: P-1, Z = 2, D_calc_ = 1.365 g cm^−3^, No. of reflection measured 3956, 2θ_max_ = 55.0°, R1 = 0.12. **Figure 1** illustrates the structure as determined. Full data can be obtained on request from the CCDC [22].

*7-Methyl-5-oxo-2-phenyl-4,5-dihydro-2H-[1,2,3]triazolo[4,5-b]pyridine-6-carbonitrile* (**5b**). Recrystallized from an EtOH/dioxane (2:1) mixture as yellow crystals, yield: 83%, m.p. above 300 °C; IR (KBr): ν/cm^−1^ 3410 (NH), 2228 (CN), 1658 (CO); ^1^H-NMR (DMSO-*d_6_*,): *δ* = 2.64 (s, 3H, CH_3_), 7.48 (t, *J* = 8.0 Hz, 1H, Ar-H), 7.58 (t, *J* = 8.0 Hz, 2H, Ar-H), 8.00 (d, *J* = 8.0 Hz, 2H, Ar-H), and 13.04 ppm (s, 1H, NH); ^13^C-NMR (DMSO-*d_6_*): *δ* = 16.94 (CH_3_), 107.37, 115.30 (CN), 119.39, 129.57, 130.41, 131.96, 139.07, 147.66, 152.55 and 160.07 ppm (Ar-C and CO); MS (EI): m/z (%) 251 (M^+^, 100), 252 (M^+^+1, 30.58). Anal. calcd. for C_13_H_9_N_5_O (251.25): C, 62.15; H, 3.61; N, 27.87. Found: C, 62.19; H, 3.55; N, 27.91.

*5-Oxo-2,7-diphenyl-4,5-dihydro-2H-pyrazolo[4,3-b]pyridine-3,6-dicarbonitrile* (**6**). Recrystallized from a EtOH/dioxane (1:1) mixture as beige crystals, yield: 80%, m.p. above 300 °C; IR (KBr): ν/cm^−1^ 3375 (NH), 2227 (br, 2CN), 1656 (CO); ^1^H-NMR (DMSO-*d_6_*): *δ* 7.64–7.66 (m, 6H, Ar-H), 7.79 (d, *J* = 7.6 Hz, 2H, Ar-H), 7.85 (d, *J* = 7.2 Hz, 2H, Ar-H) and 13.30 ppm (s, 1H, NH); ^13^C-NMR (DMSO-*d_6_*): *δ* = 100.37, 105.99, 109.67, 115.83, 124.37, 129.02, 129.91, 130.18, 130.77, 131.22, 131.71, 133.12, 135.46, 138.11, 152.78 and 159.81 ppm (2CN, Ar-C and CO); MS (EI): m/z (%) 337 (M^+^, 100), 338 (M^+^+1, 25.0). Anal. calcd. for C_20_H_11_N_5_O (337.34): C, 71.21; H, 3.29; N, 20.76. Found: 71.19; H, 3.36; N, 20.83.

*3-Benzoyl-7-methyl-5-oxo-1-phenyl-4,5-dihydro-1H-pyrazolo[4,3-b]pyridine-6-carbonitrile* (**10**).Rec- rystallized from DMSO as brown crystals, yield: 77%, m.p. 238–240 °C; IR (KBr): ν/cm^−1^ 3381 (NH), 2223 (CN), 1681, 1658 (2CO); ^1^H-NMR (DMSO-*d_6_*): *δ* 2.12 (s, 3H, CH_3_), 7.56–7.81 (m, 8H, Ar-H), 8.22 (d, *J* = 7.2 Hz, 2H, Ar-H) and 12.02 ppm (s, 1H, NH); ^13^C-NMR (DMSO-*d_6_*): *δ* 18.23 (CH_3_), 105.72, 115.34 (CN), 126.28, 127.04, 127.57, 128.59, 129.15, 129.45, 129.60, 130.07, 130.63, 133.52, 136.00, 138.95, 159.17 and 186.19 ppm (Ar-C and CO); MS (EI): m/z (%) 354 (M^+^, 100), 355 (M^+^+1, 27.1). Anal. calcd. for C_21_H_14_N_4_O_2_ (354.37): C, 71.18; H, 3.95; N, 15.81. Found: 71.24; H, 4.02; N, 15.77.

#### 3.3.3. Crystallographic Analysis for ***10***

The crystals were mounted on a glass fiber. All measurements were performed on a Rigaku R-AXIS RAPID diffractometer using filtered Mo-Kα radiation. The data were collected at a temperature of 20 ± 1 °C to a maximum 2θ value of 55.0° using the ω scanning technique. The structure was solved by charge flipping method and expanded using Fourier techniques. The non-hydrogen atoms were refined anisotropically. Hydrogen atoms were refined using the riding model.

#### 3.3.4. Crystal Data

C_21_H_14_N_4_O_2_+ one DMSO molecule, M = 354.37, monoclinic, a = 11.518(2) Å, b = 9.386(2)Å, c = 19.655(3)Å, V = 2123.5(5) Å^3^, α = γ = 90.00°, β = 92.069(7)°, space group: P2_1_/n, Z = 4, D_calc_ = 1.353g cm^−3^, No. of reflection measured 4731, 2θ_max_ = 55.0 °, R1 = 0.1088. Figure 2 illustrates the structure as determined. Full data can be obtained on request from the CCDC [23].

*3-Acetyl-7-methyl-5-oxo-1-phenyl-4,5-dihydro-1H-pyrazolo[4,3-b]pyridine-6-carbonitrile* (**14**). Recrystallized from DMF as buff crystals, yield: 80%, m.p. above 300 °C; IR (KBr): ν/cm^−1^ 3183 (NH), 2221 (CN), 1673, 1639 (2CO); ^1^H-NMR (DMSO-*d_6_*): *δ* 2.15 (s, 3H, CH_3_), 2.60 (s, 3H, CH_3_), 7.49–7.71 (m, 5H, Ar-H) and 11.91 ppm (s, 1H, NH); ^13^C-NMR (TFA-*d*): *δ* 19.82 (CH_3_), 27.25 (CH_3_), 107.63, 116.18 (CN), 125.08, 129.39, 131.18, 131.62, 132.45, 134.48, 138.03, 140.46, 154.60 and 202.41 ppm (Ar-C and CO); MS (EI): m/z (%) 292 (M^+^, 100), 293 (M^+^+1, 20.45). Anal. calcd. for C_16_H_12_N_4_O_2_ (292.30): C, 65.75; H, 4.14; N, 19.17. Found: C, 65.81; H, 4.17; N, 19.19.

### 3.4. General Procedure for the Preparation of ***15***

Independent solutions of *p*-nitrophenylacetic acid (0.9 g, 5 mmol) in Ac_2_O (5 mL) were heated in a microwave oven at 100 °C for 20 s. To these mixtures, **1a,b** (5 mmol) were added and the mixtures were heated for further 60 s at the same temperature. The reaction mixtures were cooled to room temperature the crystalline solid formed were separated by filtration and washed by cold ethanol and then hot ethanol to afford **15a,b**, respectively, as pure substances.

*N-(5-Benzoyl-2-phenyl-2H-1,2,3-triazol-4-yl)-2-(4-nitrophenyl)acetamide* (**15a**). White crystals, yield: 90%, m.p. 196 °C; IR (KBr): ν/cm^−1^ 3294 (NH), 1690, 1632 (2CO); ^1^H-NMR (CDCl_3_): *δ* 4.02 (s, 2H, CH_2_), 7.43 (t, *J* = 7.6 Hz, 1H, Ar-H), 7.50–7.62 (m, 6H, Ar-H), 7.68 (t, *J* = 7.6 Hz, 1H, Ar-H), 8.15 (d, *J* = 8.0 Hz, 2H, Ar-H), 8.27 (d, *J* = 7.6 Hz, 2H, Ar-H), 8.43 (d, *J* = 8.0 Hz, 2H, Ar-H) and 10.02 ppm (s, 1H, NH); ^13^C-NMR (CDCl_3_): *δ* 43.90 (CH_2_), 119.47, 124.05, 128.59, 128.76, 129.41, 130.41, 130.54, 132.99, 133.95, 135.98, 138.99, 141.04, 147.40, 148.35, 166.42 and 187.60 ppm (Ar-C and CO); MS (EI): m/z (%) 427 (M^+^, 56.75), 428 (M^+^+1, 15.55). Anal. calcd. for C_23_H_17_N_5_O_4_ (427.42): C, 64.63; H, 4.01; N, 16.39. Found: 64.59; H, 3.94; N, 16.43.

*N-(5-Acetyl-2-phenyl-2H-1,2,3-triazol-4-yl)-2-(4-nitrophenyl)acetamide* (**15b**). Beige crystals, yield: 87%, m.p. 207 °C; IR (KBr): ν/cm^−1^ 3358 (NH), 1686, 1641(2CO); ^1^H-NMR (CDCl_3_): *δ* 2.69 (s, 3H, CH_3_), 3.99 (s, 2H, CH_2_), 7.43 (t, *J* = 7.6 Hz, 1H, Ar-H), 7.51 (t, *J* = 7.6 Hz, 2H, Ar-H), 7.60 (d, *J* = 8.0 Hz, 2H, Ar-H), 8.12 (d, *J* = 7.6 Hz, 2H, Ar-H), 8.26 (d, *J* = 8.0 Hz, 2H, Ar-H) and 9.51 ppm (s, 1H, NH); ^13^C-NMR (CDCl_3_): *δ* 27.02 (CH_3_), 43.81 (CH_2_), 119.38, 124.06, 128.74, 129.42, 130.56, 133.67, 138.99, 140.97, 146.14, 147.43, 166.43 and 195.23 ppm (Ar-C and CO); MS (EI): m/z (%) 365 (M^+^, 27.35), 366 (M^+^+1, 6.91). Anal. calcd. for C_18_H_15_N_5_O_4_ (365.35): C, 59.18; H, 4.14; N, 19.17. Found: C, 59.24; H, 4.07; N, 19.14.

### 3.5. General Procedure for the Preparation of ***16***

Independent solutions of amides **15a,b** (5 mmol) in DMF (10 mL) containing anhydrous sodium acetate (1 g) were stirred at reflux for 1 h. Then, the reaction mixtures were cooled to rt and poured onto ice cold water. The crude products were collected by filtration, washed with water and recrystallized from the appropriate solvent to afford the corresponding azolo pyridine derivatives **16a,b** respectively.

*6-(4-Nitrophenyl)-2,7-diphenyl-2,4-dihydro[1,2,3]triazolo[4,5-b]pyridin-5-one* (**16a**). Recrystallized from an EtOH/dioxane (1:1) mixture as yellow crystals, yield: 82%, m.p. 302 °C; IR (KBr): ν/cm^−1^ 3435 (NH), 1645 (CO); ^1^H-NMR (DMSO-*d_6_*): *δ* 7.32–7.35 (m, 5H, Ar-H), 7.44 (d, *J* = 8.0 Hz, 3H, Ar-H), 7.56 (t, *J* = 7.6 Hz, 2H, Ar-H), 8.00 (d, *J* = 8.0 Hz, 2H, Ar-H), 8.10 (d, *J* = 8.0 Hz, 2H, Ar-H) and 12.90 ppm (s, 1H, NH); ^13^C-NMR (DMSO-*d_6_*): *δ* 118.62, 122.51, 128.18, 128.25, 128.89, 129.82, 129.86, 130.66, 132.29, 132.64, 133.14, 139.02, 140.68, 142.80, 146.36, 146.65 and 161.83 ppm (Ar-C and CO); MS (EI): m/z (%) 409 (M^+^, 100), 410 (M^+^+1, 30.72). Anal. calcd. for C_23_H_15_N_5_O_3_ (409.41): C, 67.48; H, 3.69; N, 17.11. Found: C, 67.51; H, 3.70; N, 17.08.

*7-Methyl-6-(4-Nitrophenyl)-2-phenyl-2,4-dihydro[1,2,3]triazolo[4,5-b]-pyridin-5-one* (**16b**). Recrysta- llized from an EtOH/dioxane (2:1) mixture as buff crystals, yield: 85%, m.p. 298 °C; IR (KBr): ν/cm^−1^ 3285 (NH), 1643 (CO); ^1^H-NMR (DMSO-*d_6_*): *δ* = 2.30 (s, 3H, CH_3_), 7.46 (t, *J* = 7.6 Hz, 1H, Ar-H), 7.58–7.63 (m, 4H, Ar-H), 8.05 (d, *J* = 8.0 Hz, 2H, Ar-H), 8.30 (d, *J* = 8.0 Hz, 2H, Ar-H), and 12.67 ppm (s, 1H, NH); ^13^C-NMR (DMSO-*d_6_*): *δ* 15.16 (CH_3_), 118.48, 122.99, 128.13, 129.84, 131.14, 131.86, 133.08, 138.08, 139.10, 142.38, 145.94, 146.81 and 161.63 ppm (Ar-C and CO); MS (EI): m/z (%) 347 (M^+^, 100), 348 (M^+^+1, 21.87). Anal. calcd. for C_18_H_13_N_5_O_3_ (347.34): C, 62.25; H, 3.77; N, 20.16. Found: C, 62.19; H, 3.81; N, 20.12.

### 3.6. General Procedure for the Preparation of ***20a-f***

Independent mixtures of 5-amino-1,2,3-triazoles **1a,b** (10 mmol), active methylene compounds **17a-c** (15 mmol) and zeolite (10% by weight) were heated at 150 °C for 1 h. Dioxane was added to the reaction mixtures followed by filtration to remove the zeolite. The crystals formed upon cooling the filtrates were collected by filtration and washed with methanol.

*Ethyl-5-oxo-2,7-diphenyl-4,5-dihydro-2H-[1,2,3]triazolo[4,5-b]pyridine-6-carboxylate* (**20a**). Pale orange crystals, yield: 76%, m.p. 205 °C; IR (KBr): ν/cm^−1^ 3391(NH), 1733, 1650 (2CO); ^1^H-NMR (DMSO-*d_6_*): *δ* 1.30 (t, *J* = 7.2 Hz, 3H, *CH_3_*CH_2_), 4.13 (q, *J* = 7.2 Hz, 2H, CH_3_*CH_2_*), 7.46 (t, *J* = 7.6 Hz, 1H, Ar-H), 7.56–7.60 (m, 5H, Ar-H), 7.65–7.67 (m, 2H, Ar-H), 8.00 (d, *J* = 7.6 Hz, 2H, Ar-H) and 13.00 ppm (s, 1H, NH); ^13^C-NMR (DMSO-*d_6_*): *δ* 13.65 (*CH_3_*CH_2_), 61.17 (CH_3_*CH_2_*), 118.81, 128.54, 128.57, 128.67, 129.86, 129.87, 130.10, 130.85, 132.11, 138.97, 140.27, 146.93, 160.14 and 165.01 ppm (Ar-C and CO); MS (EI): m/z (%) 360 (M^+^, 100), 361 (M^+^+1, 24.55). Anal. Calcd. for C_20_H_16_N_4_O_3_ (360.38): C, 66.66; H, 4.48; N, 15.55. Found: C, 66.72; H, 4.40; N, 15.49.

*6-Benzoyl-2,7-diphenyl-2,4-dihydro[1,2,3]triazolo[4,5-b]pyridin-5-one* (**20b**). Canary yellow crystals, yield: 80%, m.p. above 300 °C; IR (KBr): ν/cm^−1^ 3423 (NH), 1672, 1644 (2CO); ^1^H-NMR (DMSO-*d_6_*): *δ* 7.39–7.41 (m, 3H, Ar-H), 7.46 (t, *J* = 8.0 Hz, 3H, Ar-H), 7.52–7.62 (m, 5H, Ar-H), 7.89 (d, *J* = 8.0 Hz, 2H, Ar-H), 8.02 (d, *J* = 8.0 Hz, 2H, Ar-H) and 12.99 ppm (s, 1H, NH); ^13^C-NMR (DMSO-*d_6_*): *δ* = 118.79, 128.14, 128.45, 128.88, 129.01, 129.73, 129.89, 131.40, 131.67, 132.08, 133.17, 133.90, 136.41, 139.06, 140.49, 147.13, 161.25 and 193.91 ppm (Ar-C and CO); MS (EI): m/z (%) 392 (M^+^, 100), 393 (M^+^+1, 27.84). Anal. calcd. for C_24_H_16_N_4_O_2_ (392.42): C, 73.46; H, 4.11; N, 14.28. Found: C, 73.52; H, 4.08; N, 14.36.

*6-Acetyl-2,7-diphenyl-2,4-dihydro[1,2,3]triazolo[4,5-b]pyridin-5-one* (**20c**). Pale yellow crystals, yield: 79%, m.p. 249 °C; IR (KBr): ν/cm^−1^ 3299 (NH), 1709, 1646 (2CO); ^1^H-NMR (DMSO-*d_6_*): *δ* 2.33 (s, 3H, CH_3_), 7.47 (t, *J* = 7.6 Hz, 1H, Ar-H), 7.53-7.60 (m, 7H, Ar-H), 7.99 (d, *J* = 7.6 Hz, 2H, Ar-H) and 12.96 ppm (s, 1H, NH); ^13^C-NMR (DMSO-*d_6_*): *δ* 31.57 (CH_3_), 118.72, 128.43, 128.60, 128.96, 129.76, 129.80, 131.38, 132.13, 133.69, 138.95, 139.20, 146.64, 160.80 and 201.47 ppm (Ar-C and CO); MS (EI): m/z (%) 330 (M^+^, 100), 331(M^+^+1, 19.84). Anal. calcd. for C_19_H_14_N_4_O_2_ (330.35): C, 69.08; H, 4.27; N, 16.96. Found: C, 68.98; H, 4.35; N, 16.88.

*Ethyl-7-methyl-5-oxo-2-phenyl-4,5-dihydro-2H-[1,2,3]triazolo[4,5-b]pyridine-6-carboxylate* (**20d**). Yellow crystals, yield: 82%, m.p. 219 °C; IR (KBr): ν/cm^−1^ 3263 (NH), 1735, 1659 (2CO); ^1^H-NMR (DMSO-*d_6_*): *δ* 1.31 (t, *J* = 7.2 Hz, 3H, *CH_3_*CH_2_), 2.44 (s, 3H, CH_3_), 4.33 (q, *J* = 7.2 Hz, 2H, CH_3_*CH_2_*), 7.47 (t, *J* = 8.0 Hz, 1H, Ar-H), 7.60 (t, *J* = 8.0 Hz, 2H, Ar-H), 8.04 (d, *J* = 8.0 Hz, 2H, Ar-H) and 12.74 ppm (s, 1H, NH); ^13^C-NMR (DMSO-*d_6_*): *δ* 14.04 (*CH_3_*CH_2_), 14.31(CH_3_), 61.28 (CH_3_*CH_2_*), 118.52, 127.22, 128.33, 129.61, 129.76, 131.86, 138.90, 146.12, 159.82 and 165.08 ppm (Ar-C and CO); MS (EI): m/z (%) 298 (M^+^, 61.90), 299 (M^+^+1, 12.75). Anal. calcd. for C_15_H_14_N_4_O_3_ (298.30): C, 60.40; H, 4.73; N, 18.78. Found: C, 60.33; H, 4.84; N, 18.76.

*6-Benzoyl-7-methyl-2-phenyl-2,4-dihydro[1,2,3]triazolo[4,5-b]pyridin-5-one* (**20e**). Creamy white crystals, yield: 79%, m.p. 298 °C; IR (KBr): ν/cm^−1^ 3429 (NH), 1668, 1641 (2CO); ^1^H-NMR (DMSO-*d_6_*): *δ* 2.30 (s, 3H, CH_3_), 7.47 (t, *J* = 7.6 Hz, 1H, Ar-H), 7.55 (t, *J* = 8.0 Hz, 2H, Ar-H), 7.61 (t, *J* = 7.6 Hz, 2H, Ar-H), 7.69 (t, *J* = 7.6 Hz, 1H, Ar-H), 7.92 (d, *J* = 7.6 Hz, 2H, Ar-H), 8.06 (d, *J* = 8.0 Hz, 2H, Ar-H) and 12.75 ppm (s, 1H, NH); ^13^C-NMR (DMSO-*d_6_*): *δ* 14.18(CH_3_), 118.62, 128.34, 129.01, 129.04, 129.89, 131.90, 132.57, 134.12, 136.24, 138.83, 139.08, 146.45, 161.04 and 194.41 ppm (Ar-C and CO); MS (EI): m/z (%) 330 (M^+^, 100), 331 (M^+^+1, 22.88). Anal. calcd. for C_19_H_14_N_4_O_2_ (330.35): C, 69.08; H, 4.27; N, 16.96. Found: C, 69.15; H, 4.21; N, 17.02.

*6-Acetyl-7-methyl-2-phenyl-2,4-dihydro[1,2,3]triazolo[4,5-b]pyridin-5-one* (**20f**). Yellow crystals, yield: 86%, m.p. 242 °C; IR (KBr): ν/cm^−1^ 3435 (NH), 1694, 1648 (2CO); ^1^H-NMR (DMSO-*d_6_*): *δ* 2.36 (s, 3H, CH_3_), 2.47 (s, 3H, CH_3_), 7.44 (t, *J* = 8.0 Hz, 1H, Ar-H), 7.57 (t, *J* = 8.0 Hz, 2H, Ar-H), 8.00 (d, *J* = 8.0 Hz, 2H, Ar-H) and 12.71 ppm (s, 1H, NH); ^13^C-NMR (DMSO-*d_6_*): *δ* 13.98 (CH_3_), 30.98 (CH_3_), 118.57, 128.34, 129.79, 132.46, 133.31, 138.53, 138.95, 146.09, 160.99 and 201.93 ppm (Ar-C and CO); MS (EI): m/z (%) 268 (M^+^, 76.45), 269 (M^+^+1, 13.89). Anal. calcd. for C_14_H_12_N_4_O_2_ (268.28): C, 62.68; H, 4.51; N, 20.88. Found: C, 62.74; H, 4.47; N, 20.94.

#### 3.6.1. Crystallographic Analysis for ***20f***

The crystals were mounted on a glass fiber. All measurements were performed on a Rigaku R-AXIS RAPID diffractometer using filtered Mo-Kα radiation. The data were collected at a temperature of 20 ± 1 °C to a maximum 2θ value of 55.0° using the ω scanning technique. The structure was solved by charge flipping method and expanded using Fourier techniques. The non-hydrogen atoms were refined anisotropically. Hydrogen atoms were refined using the riding model.

#### 3.6.2. Crystal Data

C_14_H_12_N_4_O_2_, M = 268.28, triclinic, a = 4.003(4)Å, b = 13.07(2)Å, c = 13.49(2)Å, V = 643(1)Å^3^, α = 113.65(1)°, β = 91.13(2)°, γ = 94.89(2)°, space group: P-1, Z= 2, *D_calc_* = 1.385 g cm^−3^, No. of reflection measured 2935, 2θ_max_= 55.0°, R1= 0.053. **Figure 3** illustrates the structure as determined. Full data can be obtained on request from the CCDC [24].

### 3.7. N-(5-Benzoyl-2-phenyl--2H-1,2,3-triazol-4-yl)-5-oxo-2,7-diphenyl-4,5-dihydro-2H-[1,2,3]- triazolo[4,5-b]pyridine-6-carboxamide *(**22**)*

A mixture of 5-amino-1,2,3-triazole **1a** (0.66 g, 2.5 mmol), pyrazolo[4,3-*b*]pyridine **20a** (0.9 g, 2.5 mmol) and zeolite (10% by weight) in dioxane (10 mL) was stirred at reflux for 2 h, filtered to remove the zeolite, and cooled to room temperature. The solid which formed was collected by filtration, washed with ethanol, and recrystallized from dioxane giving beige crystals, yield: 68%, m.p. 271 °C; IR (KBr): ν/cm^−1^ 3404, 3314 (2NH), 1711, 1663, 1635 (3CO); ^1^H-NMR (DMSO-*d_6_*): *δ* = 7.49–7.73 (m, 14H, Ar-H), 8.01-–8.12 (m, 6H, Ar-H), 11.48 (s, 1H, NH) and 12.93 ppm (s, 1H, NH); ^13^C-NMR (DMSO-*d_6_*): *δ* 118.83, 128.52, 128.60, 128.70, 129.04, 129.75, 129.90, 130.87, 131.39, 132.26, 133.67, 136.35, 136.46, 138.61, 139.01, 140.66, 144.50, 147.02, 161.14, 162.51, and 185.99 ppm (Ar-C and CO); MS (EI): m/z (%) 578 (M^+^, 46.25), 579 (M^+^+1, 17.30). Anal. calcd. for C_33_H_22_N_8_O_3_ (578.60): C, 68.51; H, 3.83; N, 19.37. Found: C, 68.44; H, 3.87; N, 19.42.

### 3.8. (E)-Ethyl-3-(5-acetyl-2-phenyl-2H-1,2,3-triazol-4-ylamino)but-2-enoate *(**23**)*

A mixture of 5-amino-1,2,3-triazole **1b** (2.02 g, 10 mmol) and ethyl acetoacetate (1.95 g, 15 mmol) was fused at 140 °C for 20 min. The mixture was poured into water and cooled to room temperature. The crude solid which formed was collected by filtration, washed with cold ethanol, and recrystallized from ethanol to give creamy white crystals, yield: 71%, m.p. 158 °C; IR (KBr): ν/cm^−1^ 3435 (NH), 1672, 1620 (2CO); ^1^H-NMR (CDCl_3_): *δ* 1.30 (t, *J* = 7.2 Hz, 3H, *CH_3_*CH_2_), 2.50 (s, 3H, CH_3_), 2.68 (s, 3H, *CH_3_*CO), 4.27 (q, *J* = 7.2 Hz, 2H, CH_3_*CH_2_*), 4.93 (s, 1H, olefinic C*H*), 7.38(t, *J* = 8.0 Hz, 1H, Ar-H), 7.49 (t, *J* = 8.0 Hz, 2H, Ar-H), 8.05 (d, *J* = 8.0 Hz, 2H, Ar-H) and 11.95 ppm (s, 1H, NH); ^13^C-NMR (CDCl_3_): *δ* 14.55 (*CH_3_*CH_2_), 22.75 (CH_3_), 26.84 (CH_3_), 59.40 (CH_3_*CH_2_*), 92.61, 118.76, 128.05, 129.35, 133.81, 139.16, 149.00, 154.87, 168.94 and 193.51 ppm (Ar-C, olefinic C and CO); MS (EI): m/z (%) 314 (M^+^, 100), 315 (M^+^+1, 22.85). Anal. Calcd. for C_16_H_18_N_4_O_3_ (314.35): C, 61.14; H, 5.77; N, 17.82. Found: C, 61.17; H, 5.75; N, 17.86.

#### 3.8.1. Crystallographic Analysis for **23**

The crystals were mounted on a glass fiber. All measurements were performed on a Rigaku R-AXIS RAPID diffractometer using filtered Mo-Kα radiation. The data were collected at a temperature of 20 ± 1 °C to a maximum 2θ value of 55.0° using the ω scanning technique. The structure was solved by charge flipping method and expanded using Fourier techniques. The non-hydrogen atoms were refined anisotropically. Hydrogen atoms were refined using the riding model.

#### 3.8.2. Crystal Data

C_16_H_18_N_4_O_3_, M = 314.35, monoclinic, a = 4.949(2)Å, b = 14.130(4)Å, c = 23.315(7)Å, V = 1629.14(9)Å^3^, α = γ = 90.00°, β = 92.190(2)°, space group: P2_1_/n, Z = 4, D_calc_ = 1.282 g cm^−3^, No. of reflection measured 3733, 2θ_max_= 55.0°, R1= 0.0807. **Figure 4** illustrates the structure as determined. Full data can be obtained on request from the CCDC [25].

### 3.9. Ethyl-5,7-dimethyl-2-phenyl-2H-[1,2,3]triazolo[4,5-b]pyridine-6-carboxylate *(**24**)*

A solution of **23** (1.57 g, 5 mmol) in DMF (10 mL) containing anhydrous sodium acetate (1 g) was stirred at reflux for 1 h. The mixture was cooled to room temperature and poured into ice cold water. The formed solid was collected by filtration, washed with water and recrystallized from EtOH/H_2_O (2:1) to give pale brown crystals, yield: 71%, m.p. 78 °C; IR (KBr): ν/cm^−1^ 1719 (CO); ^1^H-NMR (DMSO-*d_6_*): *δ* 1.46 (t, *J* = 7.2 Hz, 3H, *CH_3_*CH_2_), 2.74 (s, 3H, CH_3_), 2.75 (s, 3H, CH_3_), 4.50 (q, *J* = 7.2 Hz, 2H, CH_3_*CH_2_*), 7.49 (t, *J* = 8.0 Hz, 1H, Ar-H), 7.57 (t, *J* = 8.0 Hz, 2H, Ar-H) and 8.40 ppm (d, *J* = 8.0 Hz, 2H, Ar-H); ^13^C-NMR (DMSO-*d_6_*): *δ* 14.27 (CH_3_), 14.71(CH_3_), 24.42 (CH_3_), 61.89 (CH_2_), 120.59, 128.72, 129.50, 129.55, 136.67, 137.78, 140.14, 154.96, 158.88 and 168.10 pm (Ar-C and CO); MS (EI): m/z (%) 296 (M^+^, 100), 297 (M^+^+1, 29.8). Anal. calcd. for C_1__6_H_1__6_N_4_O_2_ (296.33): C, 64.85; H, 5.44; N, 18.91. Found: C, 64.78; H, 5.51; N, 18.94.

## 4. Conclusions

A simple and efficient approach to the preparation of condensed pyridines, utilizing *o*-acyl heteroarmatic amines as precursors, has been developed. By using the new approach, difficulties with the classical synthesis of these substances from active methylene compounds and *o*-aminonitriles are overcome.
